# Examining Factors Influencing Colorectal Cancer Screening of Rural Nebraskans Using Data from Clinics Participating in an Accountable Care Organization: A Study Protocol

**DOI:** 10.12688/f1000research.6782.1

**Published:** 2015-07-22

**Authors:** Lufei Young, Jungyoon Kim, Hongmei Wang, Li-Wu Chen

**Affiliations:** 1 College of Nursing, Lincoln Division, University of Nebraska Medical Center, Omaha, NE, 68198, USA; 2 Department of Health Service Research and Administration, University of Nebraska Medical Center, College of Public Health, Omaha, NE, 68198, USA

**Keywords:** Screening, Colorectal Cancer, accountable care organisations, Barriers, Rural, Electronic Health Records, Mixed Methods

## Abstract

**Background:** Although mortality rates of colorectal cancer (CRC) can be significantly reduced through increased screening, rural communities are still experiencing lower rates of screening compared to urban counterparts. Understanding and eliminating barriers to cancer screening will decrease cancer burden and lead to substantial gains in quality and quantity of life for rural populations. However, existing studies have shown inconsistent findings and fail to address how contextual and provider-level factors impact CRC screening in addition to individual-level factors.

**Purpose:** The purpose of the study is to examine multi-level factors related to CRC screening, and providers’ perception of barriers and facilitators of CRC screening in rural patients cared for by accountable care organization (ACO) clinics.

**Methods/Design:** This is a convergent mixed method design. For the quantitative component, multiple data sources, such as electronic health records (EHRs), Area Resource File (ARF), and provider survey data, will be used to examine patient-, provider-, clinic-, and county-level factors. About 21,729 rural patients aged between 50 and 75 years who visited the participating ACO clinics in the past 12 months are included in the quantitative analysis. The qualitative methods include semi-structured in-depth interviews with healthcare professionals in selected rural clinics. Both quantitative and qualitative data will be merged for result interpretation. Quantitative data identifies “what” factors influence CRC screening, while qualitative data explores “how” these factors interact with CRC screening. The study setting is 10 ACO clinics located in nine rural Nebraska counties.

**Discussion:** This will be the first study examining multi-level factors related to CRC screening in the new healthcare delivery system (i.e., ACO clinics) in rural communities. The study findings will enhance our understanding of how the ACO model, particularly in rural areas, interacts with provider- and patient-level factors influencing the CRC screening rate of rural patients.

## Background

Cancer is the second most common cause of death in the US ^1^. The colorectal cancer (CRC) incidence rate for Nebraska is higher than for the US as a whole (50 for men, 37.8 for woman per 100,000 in US vs. 54.9 for men, 42.9 for women per 100,000 in Nebraska) ^1, 2^. The CRC mortality rate for Nebraska is also higher than for the US in both men (20.4 vs. 19.1 per 100,000) and women (15 vs. 13.5 per 100,000) ^1, 2^. Cancer screening plays a vital role in cancer prevention ^3^. The US Preventive Services Task Force recommends that adults aged between 50 and 75 have a CRC screening, including fecal occult blood testing (FOBT) annually, sigmoidoscopy every 5 years, or colonoscopy every 10 years ^4^. The decrease in both cancer incidence and death rates was significantly associated with the uptake of cancer screening and improved early detection ^1^. However, disparities in CRC screenings persist in rural communities ^5^. Compared to urban residents, rural residents had lower CRC screening rates (48% vs. 55%) ^6^. Remote rural residents had the lowest screening rates overall (45%) ^6^.

Studies have reported factors related to CRC screening rate in rural at three levels: patient ^5^, provider ^5, 7^ and contextual (e.g., county, rural clinics) ^8^. Patient-level barriers included social economic status ^7^, family history ^9^, access to care ^10^, comorbidity ^11^, health literacy ^5^, cost ^5^, and healthcare utilization patterns (e.g., regular physician visits) ^9, 12^. Among all the patient-level factors, receiving providers’ recommendation was one of the most commonly reported factors associated with CRC screening ^5, 7, 13– 17^. Provider-level factors influencing CRC screening were also well documented, including perceived support ^18, 19^, available time and workload ^18, 19^, attitude and belief ^20^, competing priorities ^18, 19^, and patient load ^18^. Other non-modifiable provider-level factors, such as provider’s age, gender and practice experience, also played a part in the patients’ screening behaviors ^21^. Recently, more studies have begun to examine the contextual factors, such as area poverty rate, rural clinic practice capacity, supply of rural providers (e.g., primary care physicians or specialists), which also significantly affect cancer screening behaviors ^6, 22, 23^. Despite studies conducted to address the contextual factors associated with CRC screening, the findings have been mixed as a result of variations in research design, conceptual frameworks, the use of incomplete data sources, and measurement issues ^18, 22^. For instance, Stimpson *et al*. found that the supply of specialists (e.g., gastroenterologists) is positively associated with CRC screening based on a Texas-based self-reported survey ^22^, while another study highlighted the importance of both generalists and specialists on CRC screening for the white population only, based on a single state’s Medicare claims data ^18^. The data sources used in each study (i.e., issuance claim data and/or self-report surveys) have inherent problems affecting the reliability and validity of study findings ^6, 22^. Furthermore, neither of the studies were designed to address rural specific factors related to CRC screening. As a result, these findings were contrary to what was reported in Greiner’s study ^24^ in which CRC screening among rural populations was not significantly related to the supply of physicians performing endoscopic procedures.

The interventions designed to improve rural cancer screenings have been primarily focused on overcoming patient and provider level barriers, without much consideration of contextual and delivery system level factors ^13, 15, 25– 27^. Consequently, the sustained effects of these interventions on CRC screening are uncertain. A possible explanation could be that these interventions failed to address the barriers at the healthcare system level. For instance, under the current healthcare delivery system, care providers who were paid by volume experienced high pressure to increase volume as the reimbursement rate declined, which resulted in shortened office visit time and reduced opportunities to recommend preventive services during the visit ^28^. The situation can be worse in rural clinics, where a shortage of primary care providers causes patient overload, with a large number of patient pools being covered by few clinic staff members ^29, 30^. Furthermore, without reliable data sources, such as a cancer screening registry or a state-wide electronic medical record system, it is difficult to track rural patients’ cancer screening status objectively, which further makes the evaluation of intervention effects challenging.

Accountable care organizations (ACOs) are a group of health care providers joined together to improve quality of care with lower costs ^31, 32^, by emphasizing the mechanism of care coordination, strong patient-physician relationships, use of health information technology, and value-based provider incentive systems ^32^. As a new healthcare delivery alternative, ACOs create opportunities but also challenges for rural healthcare providers ^31^. One of the requirements to become an ACO clinic is mandatory performance data tracking and reporting. This could potentially enhance patient care coordination and increase care providers’ motivation and awareness of CRC screening. However, at the same time, this could potentially increase workload for rural clinics and providers who are already stretched thin with heavy patient loads and limited resources. To date, the interaction between the new healthcare system (ACO clinics) and patient-/provider-level factors affecting CRC screening in rural populations has not been reported.

### Study purpose

The purpose of the proposed study is to examine the mechanisms of multi-level factors associated with colorectal cancer screening within the ACO context in rural Nebraska. To achieve this purpose, we have the following specific aims:

1. To identify patient-, provider-, and county-level factors influencing CRC screening of patients in rural Nebraska using data extracted from electronic health records and surveys provided by the ACO clinic providers.
2. To explore healthcare professionals’ views of barriers and facilitators of CRC screening in the ACO context, using the data collected through in-depth interviews.

### Conceptual framework

**Figure 1.**
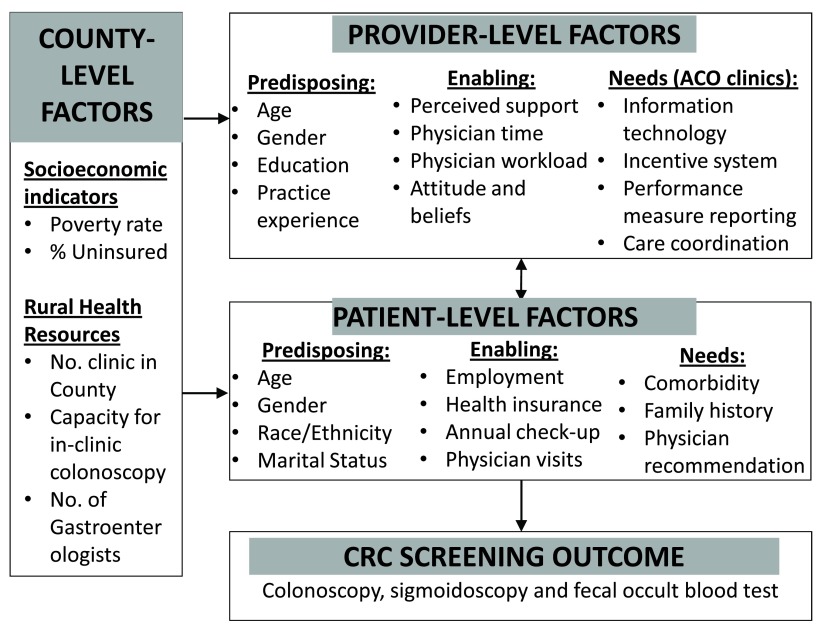
Conceptual Framework of the Proposed Study.

Based on our literature review and clinical expert input, we developed a conceptual framework derived from Gelberg-Anderson’s healthcare use behavioral model ^33^. The conceptual framework will assist in understanding rural residents’ cancer screening behavior and its correlation with individual, provider, and county level factors ( Figure 1). The model posits that cancer screening is a function of predisposing factors, enabling factors and needs at both the patient and provider levels. The model also posits that county-level factors, such as socioeconomic indicators and rural health resources, influence patient- and provider-level factors. The hypothesis illustrated by the conceptual framework will direct us in study design, variable selection, outcome measure, data collection and analysis, as well as in result interpretation.

## Methods/Design

### Study design

**Figure 2.**
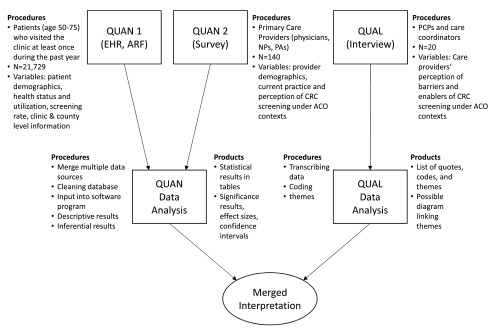
Mixed methods convergent design of the research: procedures and products.

The proposed study will use a convergent mixed method design to identify individual-, provider-, and county-level factors that influence CRC screening ( Figure 2). To address the specific aims, we will use multiple data sources including EHR, Areas Resource Files (ARF), and data collected from care provider survey and interviews ( Table 1). The study was approved by the University of Nebraska Medical Center Institutional Review Board (IRB) and all participating rural ACO clinics, receiving the number of IRB PROTOCOL # 352-15-EP.

**Table 1.** Approaches by specific aims.

Specific Aims	Domains	Data Sources	Analysis
Aim 1: Identify factors influencing CRC screening	Patient-level, provider-level, and county-level factors	EHR/ARF	Generalized mixed effects model
Aim 2: Explore care providers’ view on barriers of CRC screening in ACO context	Provider demographics, perception on barriers; ACO characteristics and its relation to CRC screening	Survey and Interview	Descriptive, Thematic coding

The quantitative analysis will answer **“what”** determines patients’ CRC screening by linking individual, provider, and system-level factors to screening outcomes, while the qualitative analysis will address **“how,”** or in what mechanism, these factors facilitate or hinder CRC screening in the ACO context. Both quantitative and qualitative data will be concurrently collected, and data will be merged during data analysis and result interpretation.

### Study setting

The study setting is a community-based ACO in rural Nebraska, which started as an advance payment ACO in the Medicare Shared Savings Program with ten independent primary care clinics, taking care of more than 14,000 Medicare patients. These clinics are located in rural counties in Nebraska and range in size from four to twelve primary care providers. All of the ACO clinics have adopted an electronic health records system with varying degrees of implementation.

***Aim 1.*** To identify patient-, provider-, and county-level factors influencing CRC screening of patients in rural Nebraska using data extracted from electronic health records and surveys provided by the ACO clinic providers.

Data source. The retrospective chart review will be conducted to obtain patient- and provider-level data from the ACO clinics using their electronic health records (EHR). De-identified EHRs will be used for data analysis. The IRB has granted a waiver of patient consent for the retrospective chart review. In addition, county-level characteristics for counties where the patients reside will be obtained from the Area Resource File, administered by U.S. Department of Health and Human Service, Health Resources and Services Administration.

Study sample. The inclusion criteria for the EHRs are: 1) the patient aged between the ages of 50 and 75 years old; 2) the patient has visited an ACO clinic at least once during the past 12 months. A total number of 21,729 patient records achieves 100% power to detect a small effect size (0.10) using a 1 degree of freedom Chi-Square Test with a significance level (alpha) of 0.05. For the provider- and county-level data, we have a total of over 50 providers including physicians, physician assistants (PAs), and nurse practitioners (NPs) from the participating clinics, providing care to over 20 counties in rural Nebraska. The expected total number of providers and counties would be sufficient to support multi-level analysis adjusting correlations at both the provider-level and the county-level. We could not find previous multi-level studies analogous to our study model. Thus, our analysis will be the first to estimate effect sizes of the explanatory variables at different levels and the random effects, which will benefit power and sample size calculation for a similar study on a larger scale in the future.

Study procedure. The research staff will work with the clinical data specialist to extract all relevant data fields for patients aged 50 to 75 years old. The dataset will be de-identified for the purpose of confidentiality and protection of patient privacy before being transferred to the researchers for analysis.

Variables. The main outcome variable of interest is whether patients are up-to-date in CRC screening, which is defined based on the US Preventive Services Task Force (USPSTF) guideline: a colonoscopy every 10 years, fecal occult blood test (FOBT) every year, or sigmoidoscopy every 5 years for adults aged 50 to 75 years old with no prior CRC and no family history of CRC. ( http://www.uspreventiveservicestaskforce.org/Page/Topic/recommendation-summary/colorectal-cancer-screening) To determine whether there are different barriers for the three types of test, we will also create three dummy variables indicating the type of test patients received for sensitive analysis. Table 2 illustrates patient, provider, and county level variables that will be included in our model. Patient-level data will be obtained from EHRs, provider-level data will be obtained second-handedly from ACO clinics through their administration or provider-survey data; county-level data will be obtained from the publicly available ARF.

**Table 2.** Factors related to Colorectal Cancer Screening.

	Factors/Variables related to screening
Patient-Level	Age, Gender, Race/Ethnicity, Marital status, Employment, County of residence, Health insurance status, Annual check-up, Number of physician visits in last year, Comorbidity, Physician recommendation
Provider-Level	Age, Gender, Race/Ethnicity, Education, Practice experience, Workload, Provider attitude and beliefs, Perceived support
County-Level (context)	County poverty rate, County uninsured rate, Number of Gastroenterologists, Number of clinics per county, In-Clinic capacity for Colonoscopy.

Analysis. We will first run descriptive statistics on all patient characteristics: (1) mean and standard deviation are used to report continuous variables; (2) frequency and percentage are used to report categorical variables. Chi-square tests will then be performed to examine if there are statistically significant differences in each of the patient characteristics between those up-to-date on CRC screening and those not up-to-date. To account for the correlation among patients clustered with provider and county level, a generalized linear mixed effects model will be used to examine the simultaneous effects of all patient-, provider- and county-level characteristics on CRC screening after controlling for other characteristics. This study is the first to control for correlations at two cluster levels when examining the factors influencing CRC screening. Fixed Effects model and Random Effects model will both be conducted to examine the mechanisms that link factors to screening outcomes and the interaction between different levels. SAS version 9.2 will be used for data analysis.

***Aim 2.*** Identify healthcare professionals’ view of the challenges and opportunities of CRC screening under ACO context.

Data source. The research team will use semi-structured surveys and in-depth interviews. The two methods will be used in parallel to triangulate methodological weaknesses of self-administered surveys and in-depth interviews.

Study sample. The inclusion criteria for participants for Aim 2 are health care professionals working in rural ACO clinics. Healthcare providers are defined as physicians, PAs, NPs, nurses, and care coordinators.

Study procedure. *a) Survey.* A paper-and-pencil, self-reported survey will be distributed to healthcare professionals working in ACO clinics, including physicians, PAs, NPs, nurses, and care coordinators. The survey questionnaire will be developed by the research team in collaborating with ACO partners as a part of ACO’s annual continuing medical education program. The survey will assess healthcare professionals’ knowledge, attitude, practice pattern, and perceived barriers of CRC screening, as well as delivery system characteristics (e.g., ACO) that influence CRC screening. A combination of closed-ended and open-ended question will be used. The pilot testing will be conducted to assess the content validity and reliability of the tool. The survey data will be analyzed using SPSS version 22 ^34^. Descriptive analysis will be used to illustrate care provider characteristics, provider enabling factors and needs, and ACO characteristics related to CRC screening.

*b) In-depth interview*. In parallel with the survey, the research team will conduct interviews with 15–20 key informants, including two or three persons from each professional role in the ACO setting: physicians, PAs, NPs, administrators, nurses, and care coordinators. The research team will use the combination of convenient and purposive sampling, as different professional roles (e.g., administrator or physician) will provide unique aspects about provider and delivery system level factors under the ACO context. Interviewees will be asked about their perception of barriers and facilitators of CRC screening under rural ACO contexts, as well as their opinion of how ACO model is interacting with the promotion of CRC screening ( Table 3).

**Table 3.** Interview guideline.

Barriers and facilitators	1. How do you communicate with your patients about CRC screening? 2. What do you think are barriers or facilitators of CRC screening? 3. What makes it difficult for you to promote CRC screening in rural practice?
Delivery system-level factors	4. How do you view the ACO model in relation to cancer prevention? 5. In what way can the ACO model influence promotion of cancer screening in rural areas? 6. Are there any other challenges (or opportunities) that you have felt in relation to the ACO model? 7. Can you think of additional ways we can help ACO care providers to promote cancer screening?

*c) Recruitment and data collection mode.* The research team will attend regular ACO board meetings and care-coordinator meetings to identify and recruit key informants for interviews. Invitation letters and flyers will also be used to raise awareness of the study and to promote participation rate. Face-to-face or telephone interviews will be conducted depending on the preference of interviewees. The interview will be 30 to 35 minutes in length and will be audio-recorded and transcribed by experienced and professionally trained research staff. A cross-validation of the interview transcript will be conducted by the research team. Table 4 lists the interview protocol regarding the IRB, compensation, interview plan, and confidentiality.

**Table 4.** Interview Protocol.

IRB	IRB approval will be attained from UNMC and a cover letter expressing research goals, procedures, potential benefits, and risks will be developed by the research team and provided to the participants.
Compensation	Each interview participant will receive incentives for their time and expertise.
Interview Plan	The interview guide will be developed and reviewed by the research team and expert panels prior to dissemination. At least two investigators will participate in the interview. One person will ask questions and another person will take notes. Interviewer training will be completed prior to the interview.
Confidentiality	All interview data will be stored in password protected computers and UNMC secure servers and will not be shared with any other person outside of the research team, except for academic publication. Individual identifiers will not be revealed in publication.

Analysis. Data will be analyzed by inductive (ground-up) and deductive development and organization of thematic codes. Using the notes taken by the researchers and literature review, the research team will develop a coding structure, which includes key conceptual domains and participant perspectives. Minor modifications will be made iteratively until the model is saturated. Data will be coded and analyzed using NVivo qualitative analysis software (QSR NVivo 10) ^35^.

### Data management and result dissemination

Data management protocol has been developed for this study, including guidelines and procedures for data collection, validation, entry, storage, analysis and dissemination. All study data will be stored in the Research Electronic Data Capture (REDCap) database ( http://www.project-redcap.org/). REDCap is a reliable and secure web-based application that allows for comprehensive management of the data collection process that is supported at University of Nebraska Medical Center and University of Iowa. Study participants will have access to de-identified data. The results of this study will be disseminated through publications and presentations. The dataset be provided for public and statistical use.

## Discussion

To our knowledge, this will be the first study quantitatively and qualitatively examining multi-level factors influencing CRC screening in the new healthcare delivery system (i.e., ACO clinics) in rural communities. The evidence of how the new rural ACO clinics interact with county-, provider- and provider-level factors and the combined effects on cancer screening is missing in rural settings. Our study will fill these knowledge gaps through a two-step approach using clinic-level data: 1) quantitatively examine multi-level factors influencing CRC screening in rural adults between age 50 and 75 receiving care from ACO clinics; 2) qualitatively explore factors related to CRC screening guided by the findings from quantitative data. Results from the proposed study will provide practice and managerial implications to the field by helping ACO clinicians and administrators to best utilize ACO infrastructures, such as care coordination, health information technology, value-based incentive system, and reporting of performance measures, to promote cancer screening of rural patients.

In addition, the study will address the problems with current literature in terms of the inconsistent findings, limitations in data sources, and missing evidence related to CRC screening within the context of the new rural healthcare system. Given that effective and sustained interventions require strategies aligning provider- and patient-level factors with care delivery system and community characteristics ^28^, the findings will help identify and develop strategies to target multi-level factors related to CRC screening in rural areas. Furthermore, the study will provide managerial implications for the operations of ACO organizations and impact policy changes in rural settings.

### Research implication

The project will help develop the practice-based research network between an academic setting and rural ACO clinics in Nebraska to promote cancer screening. If feasible and sustainable, we will continue to build a larger scale cancer research initiative, as well as extend to other practice-driven research programs (e.g., obesity related cancer prevention and control, interventions to manage cancer in patients with competing co-morbidities, and house-call programs for cancer patients living with complex complications, etc.).

### Education and practice implication

The partnership between academic and rural ACO clinics will help identify the clinic sites and capstone project topics for students; while the clinics can utilize academic resources to conduct mandatory performance improvement projects and measure tracking.

## Conclusion

Eliminating barriers to CRC screening could lead to substantial gains in quality and quantity of life and decrease the CRC burden on public health; however, sustained and effective interventions to promote screening remain uncertain. Our study will help determine the mechanism of effective intervention to optimize CRC screening by qualitatively and quantitatively examining the impact of multi-level factors on CRC screening in rural communities. To explore the additional data resources, we will use clinic data and ACO clinic electronic health records to conduct our study. If successful, our findings will add evidence and inform the design of effective interventions tailored to promote cancer screening in rural populations.
