# Supplementary material for: Examining Factors Influencing Colorectal Cancer Screening of Rural Nebraskans Using Data from Clinics Participating in an Accountable Care Organization: A Study Protocol
Source: F1000Res. 2015 Jul 22;4:298. [Version 1] doi: 10.12688/f1000research.6782.1 (PMC4544372; doi:10.12688/f1000research.6782.1)
Supplement: Peer review history

# Peer review: Referee response for version 1

**DOI:** 10.5256/f1000research.7286.r9595

**Published:** 2015-08-04

**Authors:** Paul Terry

**Affiliations:** 1 Department of Public Health, University of Tennessee, Knoxville, TN, USA

1. It would have been interesting to learn why the authors believed the ACO patient population might be unique in terms of their barriers to CRC screening.  Given the many other studies cited, were there any hypotheses driving the study other than this ACO is a relatively new healthcare organization? 
2. The first mention of “sensitive analysis” under “Study Setting” is unclear.
3. Effect sizes and variability will not be generalizable to other populations, will they?  It seems that the authors may have implied this, unless they are planning to address the same issues in a future larger study in rural Nebraska.   If this latter is the case, then this is a pilot study?  Given the size and statistical power, I would think this is not a pilot study.  Hence, the scope of the present study was not totally clear. 
4. It is unlikely that this study alone will effectively address the mixed results from previous studies.  At least the authors might try to justify this further and suggest other necessary components of reconciling the disparate findings. 
5. It is not entirely clear how the data will be used to increase CRC screening.  What has been the experience of previous investigators who have attempted to use data about “barriers to CRC screening” to increase screening in their populations?

I have read this submission. I believe that I have an appropriate level of expertise to confirm that it is of an acceptable scientific standard.

# Peer review: Referee response for version 1

**DOI:** 10.5256/f1000research.7286.r9594

**Published:** 2015-08-04

**Authors:** Young Dae Kwon

**Affiliations:** 1 Department of Humanities and Social Medicine, Catholic University of Korea, Seoul, Korea, South

This research proposal has dealt with interesting topic, and expects to conduct a comprehensive and systematic study that is able to understand the factors associated with CRC screening of rural area residents based on this. Especially, I think it is great meaningful in that it will carry out with quantitative and qualitative approach together. The overall configuration is well described, but there are minor problems to be improved.

- The authors described that " *Existing studies have shown inconsistent findings and fail to address how contextual and provider-level factors impact CRC*" in the Background of the Abstract. However, Background in the main manuscript could not clearly show and organize that there is no consistency in the existing research results in detail.
- In Background, It would be better to explain how considering the patient, provider and county level factor together could improve CRC screening rate or why the comprehensive consideration is so important in CRC screening rate improvement.
- " *No. Clinic in County’* in Figure1 seems better to be modified as ‘ *No. of Clinics in County’.*
- In Figure 1, the education variable is included only on the provider factor, but I think it is also an important variable as the patient factor.
- There are only two quantitative variables in socio-economic indicator of county level: 'poverty rate' and '% uninsured'. Are these two variables sufficiently able to reflect unique characteristics of the rural area?
- It seems to require a detailed explanation for the abbreviations used in the Figure 2.
- I think the biggest advantage and characteristic of this study is to perform quantitative approach and qualitative approach together. It would be better to describe in detail how you will conduct integration and linking of two research methods. Instead of just a parallel listing, specific plans for organic connection and interpretation of the results from two methods should be presented.

I have read this submission. I believe that I have an appropriate level of expertise to confirm that it is of an acceptable scientific standard.
